# Clinical Benefit of Autologous Platelet-Rich Plasma Infusion in Ovarian Function Rejuvenation: Evidence from a Before-After Prospective Pilot Study

**DOI:** 10.3390/medicines10030019

**Published:** 2023-02-27

**Authors:** Athanasios Garavelas, Panagiotis Mallis, Efstathios Michalopoulos, Eros Nikitos

**Affiliations:** 1 Institute of Life, IASO Maternity Hospital, 37-39, Kifissias Avenue, 151 23 Athens, Greece; 2 Hellenic Cord Blood Bank, Biomedical Research Foundation Academy of Athens, 4 Soranou Efessiou Street, 115 27 Athens, Greece

**Keywords:** ovarian insufficiency, platelet-rich plasma, FSH, AMH, follicles, growth factors, menopause, hormone therapy

## Abstract

**Background**: The intraovarian administration of autologous platelet-rich plasma (PRP) acts beneficially for the stimulation of follicle production in women presenting different forms of ovarian dysfunction. This pilot study aimed to evaluate and provide significant data regarding the efficacy of PRP to rejuvenate the ovaries. **Methods:** A total of 253 women aged 22–56 years, were divided into five groups, based on their status. All participants signed for informed consent for the current study. Blood sampling, preparation of PRP and intraovarian infusion of the latter were performed on all participants. The evaluation of PRP efficacy, a two-month follow-up detecting the levels of follicle-stimulating hormone (FSH), luteinizing hormone (LH), estradiol (E2) and anti-mullerian hormone (AMH), was performed for all participants. For women with advanced ages (>48 years), the restoration and regularity of the menstrual cycle were additionally evaluated. **Results:** After the two-month follow-up, the majority of the participants presented improvement in their hormonal profiles. Additionally, 17% of the women in this pilot study successfully conceived. The restoration of the menstrual cycle was detected in 15% of the women with advanced ages. **Conclusions:** Intraovarian infusion of autologous PRP exhibited remarkable evidence and promising results to restore ovarian insufficiency.

## 1. Introduction

Ovarian dysfunctions, such as premature ovarian insufficiency (POI) and poor ovarian response (POR) are conditions characterized by the collapse of ovarian function and are currently considered as a global public health issue [1,2,3,4]. POI represents a rare gynaecological condition, characterized by disturbances of the menstrual cycle and infertility. The golden standard treatment for POI is currently considered to be the use of hormone replacement therapy (HRT), however, this treatment is accompanied by a high percentage of failure in restoring ovarian function [5]. On the other hand, POR refers to a situation where a low number of oocytes are retrieved after ovarian stimulation, with exogenous administrated hormones. Current treatments for POR exhibit a low success rate, thus making it harder for candidate parents to genetically acquire offspring [6]. Besides these conditions, women also in perimenopause and menopause stages, which have postponed their pregnancies due to the modern way of life or other existing factors (e.g., socioeconomic status), still may want to conceive [7]. For these reasons, it is estimated that annually, in developed countries, more than one million cycles for in vitro fertilization (IVF) are performed [8]. Notably, in Greece, more than 300,000 couples are considered to have fertilization issues and more than 14,000 cycles for IVF are performed each year, since 2013 [9,10].

Based on European Statistics, the average age for women to give birth to their first child has increased from 23 to 30.7 years [11]. Primary factors that are associated with female infertility include endocrine dysfunction, failure of embryo implantation, endometriosis, and other related pathologies such as polycystic ovary syndrome (PCOS), various environmental factors and inflammatory disease [12]. Considering the natural oocyte elimination due to the ageing process, in combination with the above data, more women have difficulties in conceiving a child [13]. For this purpose, the rejuvenation of the ovarian function may result in increased release of follicles from the available reservoir and can potentially increase the possibility of a successful pregnancy. However, to restore ovarian folliculogenesis utilizing exogenous administrated factors comprises a highly demanding task.

Ovarian folliculogenesis is divided into gonadotropin-independent and gonadotropin-dependent stages [14,15,16]. Early in this process the development of pre-antral follicles from primordial follicles (oocytes surrounded by a single layer of flattened granulosa cells) can be driven through the utilization of intraovarian factors [14,15,16]. Specifically, primordial follicles give rise to primary follicles and then to secondary follicles. Then, the production of pre-antral follicles is performed [17]. The pre-antral phase is stimulated by the FSH, which leads to the production of antral follicles [18]. Antral follicles are composed of the oocyte, the granulosa cells (which are further classified into cumulus and mural granulosa cells) and theca cells (classified into internal and external theca cells) [14,15,16]. However, the hormone responsible for the maturation of antral follicles is LH. LH is under the control of the gonadotropin-releasing hormone (GnRH) secreted by the hypothalamus [19]. It has been shown that FSH stimulates the production of LH receptors (LHR) in granulosa cells [20]. The interaction between LH and its receptors leads to the activation of adenyl-cyclase, and subsequently the production of cyclin Adenosine Monophosphate (cAMP). In this way, antral follicles reduce their response to FSH by increasing the number of LHR. Additionally, AMH plays an important role in the production of antral follicles and is exclusively produced by the granulosa cells, during folliculogenesis [20]. Finally, at the maturation step of the antral follicles that will finally ovulate, the levels of AMH and estrogen (E2) are increased, while at the same time, the levels of FSH and LH are decreased [14,15,16]. AMH is considered one of the crucial regulators of follicle growth and development. Besides its contribution to the maturation of antral follicles, AMH also inhibits the recruitment of primordial follicles from the resting pool, while in parallel can modify the growth of preantral and antral follicles by impairing the follicles’ interaction with the FSH.

However, the majority of follicles undergo atresia, a process dependent on apoptotic and survival stimuli cues. In this process, a set of cytokines, such as the tumor necrosis factor (TNF) and IL-1 are responsible for follicle apoptosis initiation [21].

Ιn the ovarian folliculogenesis, growth factors such as the transforming growth factor—β (TGF-β), fibroblast growth factor (FGF), vascular endothelial growth factor (VEGF), insulin-like growth factor (IGF) and others positively influence the transition from primordial to pre-antral follicles [22]. In addition, it is known that the TGF-β can influence the expression of the FSH receptor (FSHR), thus this interaction (between FSH-FSHr) provides further survival stimuli for the antral follicles [23]. In the same way, TGF-β favors the expression of the LH receptor (LHR), where LH and progesterone can inhibit follicular apoptosis. Moreover, TGF-β, with the above-mentioned growth factors, such as IGF, VEGF and FGF, have a significant role in the regulation of follicular growth and maturation [23].

Considering the above data, rejuvenation of the ovarian function using the intraovarian injection of exogenous growth factors may comprise an alternative therapeutic strategy, to enhance the possibility of pregnancy. In this context of ovarian rejuvenation, lately, the use of autologous PRP has been extensively used in a great number of wound healing and tissue regeneration approaches [24,25,26]. PRP is considered a rich source of growth factors, released from the a-granules of platelets, upon utilizing freeze-thawing cycles or the administration of platelet activation factors, such as baxotrobin or calcium gluconate [27,28]. The released growth factors can interfere with the aforementioned hormone receptors and can activate specific signaling pathways [29].

In the past, endometrial infusion of PRP resulted in proper management of chronic endometriosis [30,31]. Nowadays, the utilization of PRP-derived growth factors has been reported in an attempt to restore ovarian function in women suffering from POR, POI or at perimenopause and menopause stage [32,33,34,35]. The majority of the studies utilizing the autologous PRP for ovarian rejuvenation present encouraging results, still the intraovarian injection of PRP must be further evaluated, to assure safer conclusions.

For this purpose, the aim of this study was the evaluation of the intraovarian injection in women with ovarian dysfunction due to POR, POI, perimenopause and menopause, in terms of ovarian rejuvenation, which may lead to an increased release of follicles from the available reservoir. To investigate the beneficial impact of PRP in ovarian function, specific biochemical parameters will be determined including FSH, LH, AMH and E2. Data obtained from this study may reflect significant evidence in order to better understand the effect of PRP both at the cellular and molecular level, understanding better its positive effect in ovarian function restoration.

## 2. Materials and Methods

### 2.1. Study Design

The current study was performed between May 2018 and December 2021 at the Institute of Life, IASO hospital. Athens, Greece. The primary aim of this study was to evaluate the effect of the intraovarian infusion of autologous PRP to restore ovarian function. This study was designed as a randomized prospective observational pilot study that was approved by the Scientific Board of “IASO” Maternity Hospital with Registration Number: 10/10/19. The whole study followed the regulations as outlined by the declaration of Helsinki, and also those outlined by the Greek Bioethics Committee of Human Reproduction. In addition, informed consent was signed by all participants of this study. Initially, a total of 582 participants were interested in participating in the current pilot study. Then, the classification of the participants based on the Bologna criteria as provided by the European Society of Human Reproduction and Embryology (ESHRE) was performed into the following groups: Group A (22–38 years), Group B (39–44 years), Group C (45–57 years), Group D (48–50 years) and Group E (51–56 years). A detailed description of the participants’ characteristics can be found in Table 1.

### 2.2. Exclusion Criteria

General exclusion criteria included the presence of autoimmune disorders, other chronic inflammatory diseases, sexually transmitted diseases and infectious diseases, tubal factor infertility, tubal obstruction, thyroid dysfunction, endometriosis, hematological disorders (e.g., anemia, thrombophilia disorders, hematological cancers), cardiovascular disease, and body mass index (BMI) >30 or <18.5, and presence of gynaecological cancer or familiar history for gynaecological cancer. Women who presented one or more of the above criteria were generally excluded from this study.

### 2.3. Examination before the PRP Intraovarian Administration

The standard examination for the reproductive dynamic of the participants enrolled in this study, included the evaluation of FSH, LH, AMH and E2. The determination of these biochemical markers was performed on day 3 of the menstrual cycle in groups A and B, while in groups C-E, was performed on a random day.

The quantification of FSH, LH, AMH and E2 was performed using the chemiluminescent microparticle immunoassay (Roche Diagnostics GmbH, Mannheim, Germany) using the Roche analyzer (Roche Cobas 4111, Basel, Switzerland).

### 2.4. Preparation Protocol for PRP

Initially, blood sample collection was performed from all participants of the study, on the same day that the intraovarian infusion was performed. The PRP production protocol relied on the kit of Acronnyx (Abioplex, Euston, London, UK), and the whole procedure was performed according to the manufacturer’s instructions. For PRP production, an initial blood volume of 60 mL was collected from the participants, followed by a two-step centrifugation process. Finally, the produced autologous PRP could either be immediately administrated or stored for a maximum of 2 h at 4 °C.

### 2.5. PRP Intraovarian Infusion Methodology

To evaluate the impact of PRP on ovarian function restoration, participants who received HRT were invited to discontinue it for at least 6 months prior to the initiation of this study. The PRP intraovarian infusion was assisted using transvaginal ultrasound monitoring. Then, in each ovary, an intramedullary injection of PRP was performed on multiple sites with a 17-gauge single-lumen needle. After the ovary penetration, 4 mL of PRP was gradually infused. After the PRP infusion, an ultrasound examination was performed to check the total vascular integrity of the pelvis. Then, the participants remained in the supine position for 15 min, before leaving the hospital.

### 2.6. Follow-Up Monitoring

The evaluation of the effectiveness of the PRP treatment in ovarian function recovery involved two months of follow-up monitoring. The assessment of the ovarian function involved the quantification of FSH, LH, AMH and E_2_, for two constitutive menstrual cycles. The levels of FSH, LH, AMH and E_2_ were performed on day 3 of the menstrual cycle, as has been described earlier in this article.

For groups A and B that followed the PRP infusion, the outcome was positive when these participants failed to be re-established as POR, POI or pre-menopause.

**Figure 1:**
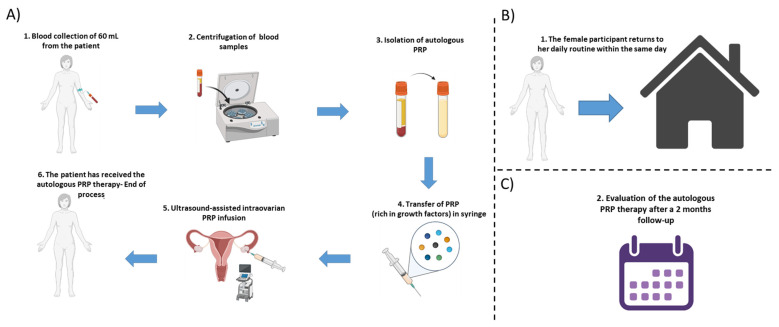
Schematic workflow of the autologous PRP methodology. (**A**) Initially a blood collection of 60 mL is performed from each participant, followed by the centrifugation step and the isolation of PRP. The isolated PRP was loaded into a 5 mL syringe and finally, the ultrasound-assisted intraovarian infusion of PRP is performed to the patient. (**B**) After the PRP administration, the female participant can return to her daily routine, within the same day. (**C**) The assessment of the autologous PRP therapy was monitored by a 2-month follow-up.

For groups D to E, the positive outcome after the PRP infusion was considered the restoration of the menstrual cycle. Furthermore, the regularity of the menstrual cycle was also evaluated. The menstrual cycle regularity was defined as less than seven days difference between two consecutive cycles. In the same way as above, the quantification of FSH, LH, AMH and E_2_ was performed on the 3rd day of the menstrual cycle. The schematic workflow of the PRP methodology is represented in Figure 1. In addition, ovarian ultrasound monitoring was performed to all patients before and after the autologous PRP administration.

### 2.7. Statistical Analysis

The current investigation represented a randomized observational uncontrolled pilot study, which included 5 groups.

The statistical analysis of this study was performed using GraphPad Prism v. 6.0.1 (GraphPad Software, San Diego, CA, USA). All data were analyzed using parametric tests such as *t*-test and ANOVA. Statistically significant differences between group values were considered when the *p* value was less than 0.05. Indicated values were presented as mean ± standard deviation.

## 3. Results

### 3.1. Assessment of the Intravorian Infusion of PRP

**Figure 2:**
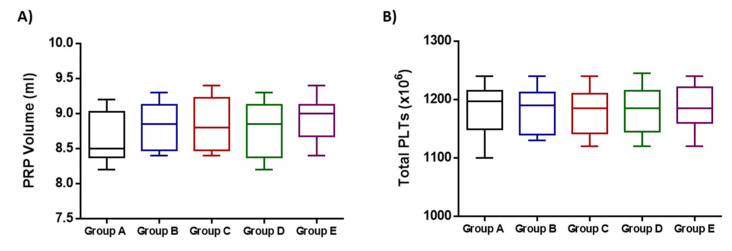
Quality characteristics of the obtained PRP from the participants of the study. (**A**) Determination of PRP volume of all participants. (**B**) Determination of the total PLT number of all participants. No statistically significant differences were observed either in PRP volume (*p* = 0.696) or total PLTs (*p* = 0.999) between all participants of this study.

The preparation of autologous PRP was successfully performed for all participants. To ensure the quality characteristics of the produced PRP, volume and total PLTs were determined in all study groups. Specifically, the PRP volumes of groups A-D were 8.7 ± 0.3 mL, 8.8 ± 0.3 mL, 8.7 ± 0.4 mL and 8.9 ± 0.3 mL, respectively (Figure 2). The total PLT numbers of groups A-E were 1186 ± 41 × 10^6^, 1182 ± 37 × 10^6^, 1178 ± 38 × 10^6^, 1183 ± 39 × 10^6^, 1187 ± 38 × 10^6^ (Figure 2). No statistically significant difference regarding the obtained PRP volume (*p* = 0.696) and total PLT number (*p* = 0.999) was observed between all groups.

### 3.2. Evaluation of the Intraovarian PRP Infusion Outcome in All Participants

**Figure 3:**
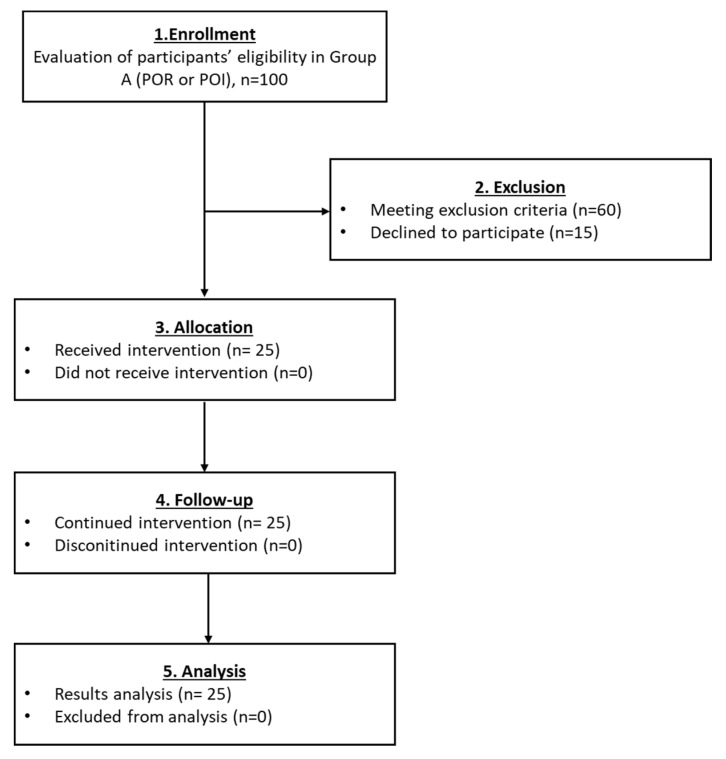
The flowchart of Group A presents enrollment, allocation, follow-up and analysis of all participants.

**Figure 4:**
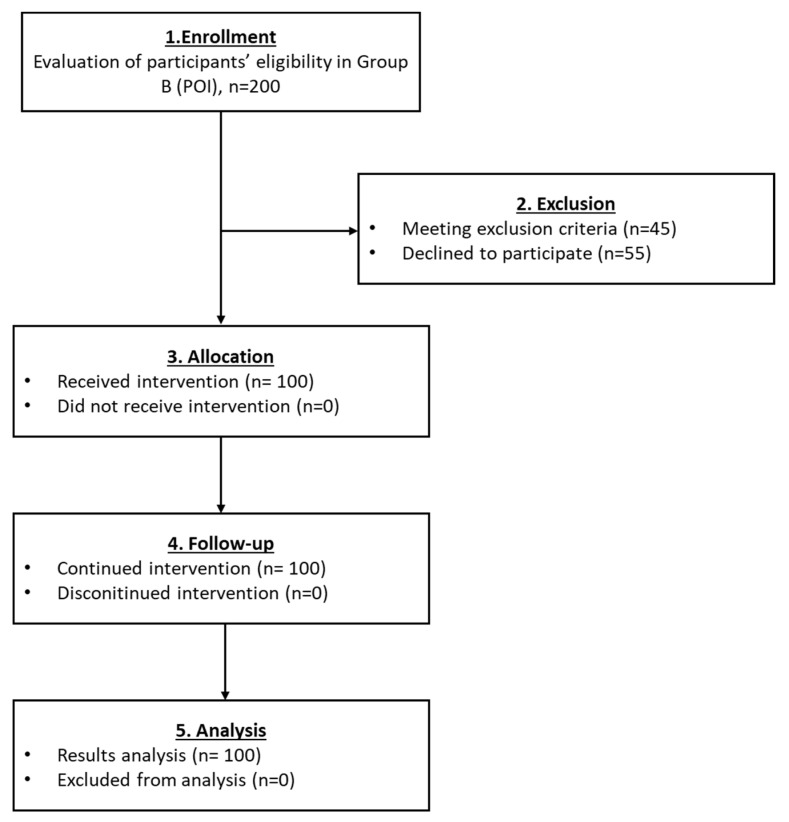
The flowchart of Group B presents enrollment, allocation, follow-up and analysis of all participants.

**Figure 5:**
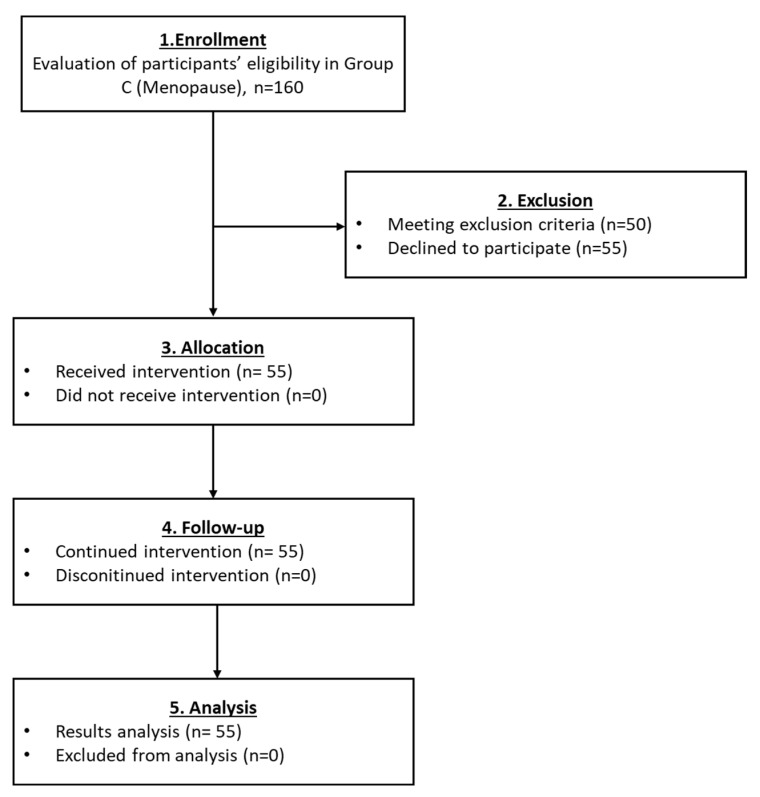
The flowchart of Group C presents enrollment, allocation, follow-up and analysis of all participants.

**Figure 6:**
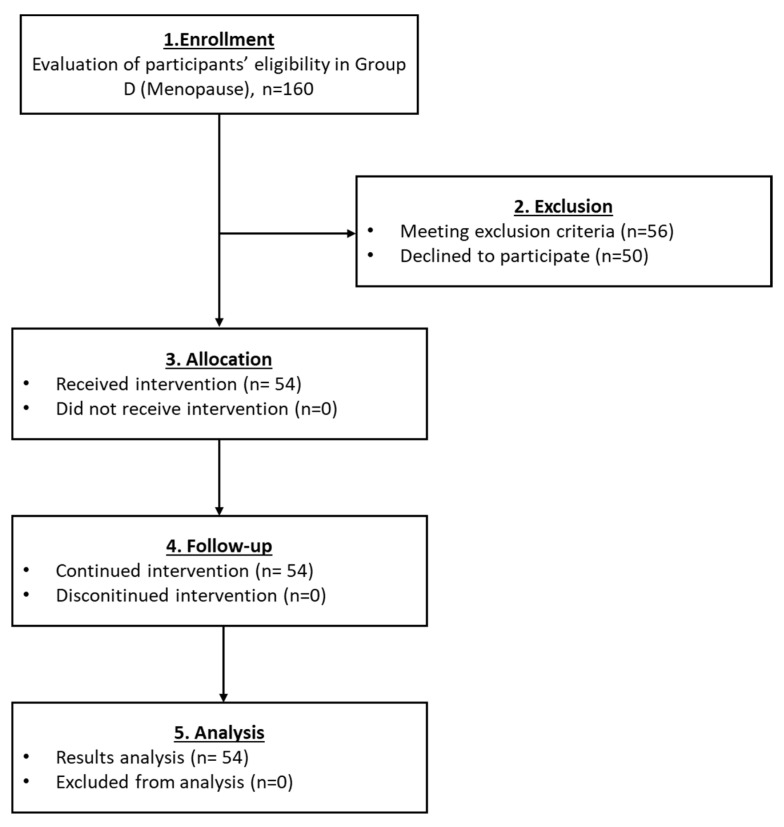
The flowchart of Group D presents enrollment, allocation, follow-up and analysis of all participants.

The current pilot study was initiated between May 2018 and December 2021 and involved the enrollment of 582 participants aged 22–56 years, who presented POI, POR, perimenopause and menopause. From those, only 25 women met the inclusion criteria, were classified into five groups and participated in the proposed pilot study. A detailed description of the workflow of each group is provided in Figure 2, Figure 3, Figure 4, Figure 5 and Figure 6. The results of this study showed a decrease in FSH and LH levels and in parallel the elevation of E2 and AMH after the first month of the follow-up. The same outcome was maintained after the second month of the follow-up in the majority of the participants.

#### 3.2.1. Results Regarding the Participants of Group A

Specifically for group A (POR or POI), a total of 25 women were finally eligible to participate (Figure 3). A decrease of more than 50% was detected for the FSH and LH whereas, the levels of E2 and AMH were elevated by more than 44% and 31%, respectively, in all participants (Table 2). Moreover, statistically significant differences regarding the levels of FSH (*p* = 0.001), LH (*p* = 0.046), E2 (*p* = 0.035) and AMH (*p* = 0.012), were observed after the first and second month of follow-up (Table 2 and Figure 8). In addition, 6 of the participants after the PRP infusion became pregnant and gave successful live births. Ultrasound proofs regarding the beneficial effect of the autologous PRP administration are provided as Supplementary Materials (Figure S1).

#### 3.2.2. Results Regarding the Participants of the Group B

Regarding group B, a total of 100 participants were finally included (Figure 4). After the PRP infusion, a statistically significant decrease in FSH (*p* < 0.001) and LH (*p* = 0.001) levels were observed in all participants of the current group. In parallel, the elevation of E2 and AMH levels was observed (Table 3 and Figure 8). Moreover, 28% of the participants with diagnosed POI, after the PRP infusion, achieved successful pregnancies and live births. Ultrasound proofs regarding the beneficial effect of the autologous PRP administration are provided as Supplementary Materials.

#### 3.2.3. Results Regarding the Participants of Group C

In group C, a total of 55 women with menopause successfully met the inclusion criteria and finally enrolled (Figure 5). After the PRP infusion, a reduction in FSH and LH levels with a parallel increase in E2 and AMH levels was detected (Table 4 and Figure 8). However, statistically significant differences were detected only in the levels of FSH and AMH (*p* < 0.001), while the levels of LH and E2 did not present any statistically significant difference. Besides the variance in the above levels, 18% of the participants reported the return of their menstrual cycle and 13% (from the 55 women) achieved pregnancies after IVF. In total, 7 normal live births were reported in this group (Table 4). Ultrasound proofs regarding the beneficial effect of the autologous PRP administration are provided as Supplementary Materials.

#### 3.2.4. Results Regarding the Participants of Group D

The number of participants who finally enrolled in group D was 54 (Figure 6). The levels of FSH and LH slightly declined after the PRP infusion. Furthermore, the levels of E2 and AMH were increased (Table 5 and Figure 8). A statistically significant difference was observed only in the levels of E2 (*p* = 0.010). Return of the menstrual cycle was observed in 9 out of 55 women, and also 2 women were able to become pregnant and give a normal birth (Table 5). Ultrasound proofs regarding the beneficial effect of the autologous PRP administration are provided as Supplementary Materials.

#### 3.2.5. Results Regarding the Participants of Group E

**Figure 7:**
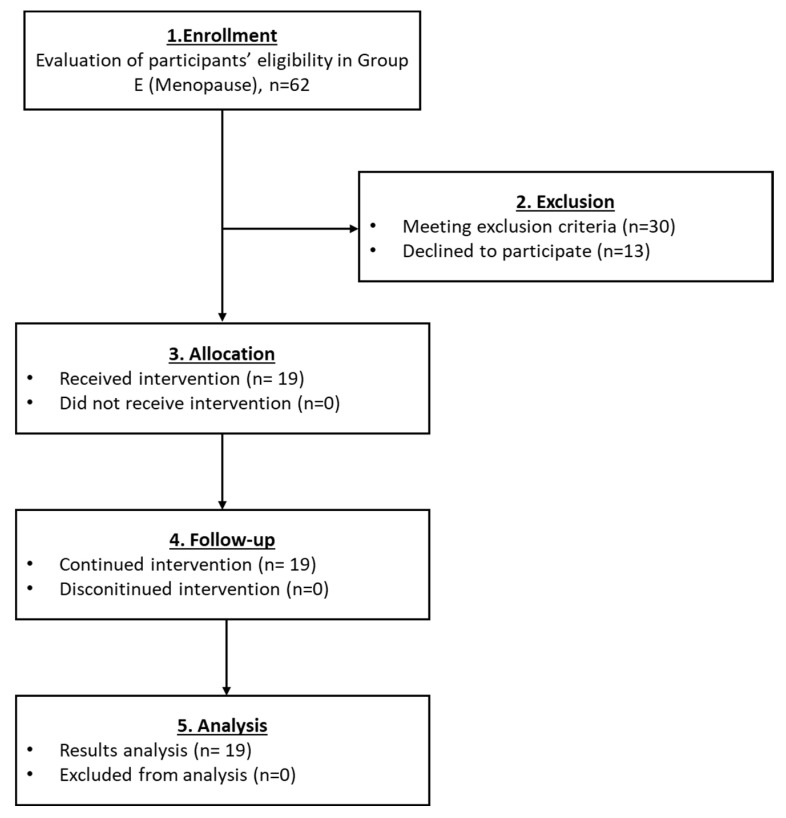
The flowchart of Group D presents enrollment, allocation, follow-up and analysis of all participants.

**Figure 8:**
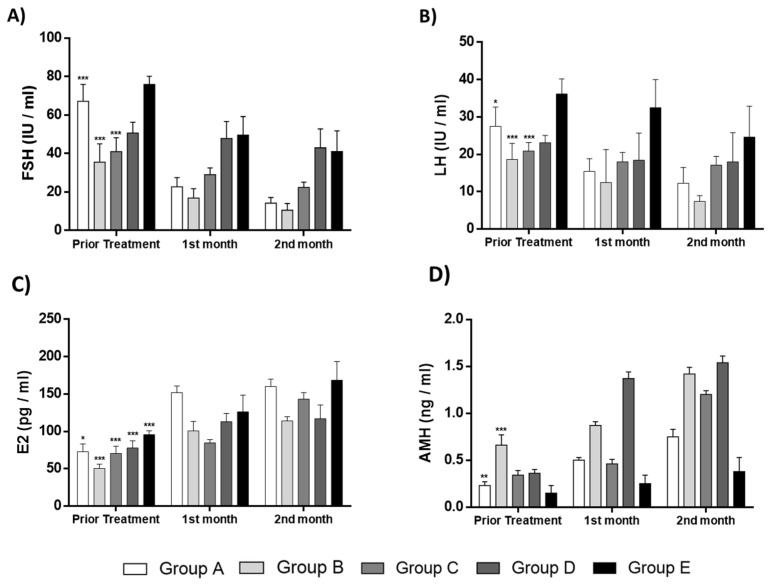
Representable diagrams regarding the levels of FSH, LH, E2 and AMH of the current pilot study. (**A**) FSH levels of all groups prior treatment, after the 1st and 2nd month follow-up. Statistically significant differences were observed in FSH levels before and after the PRP administration in the groups A (*p* < 0.001), B (*p* < 0.001) and C (*p* < 0.001). (**B**) LH levels of all groups prior treatment, after the 1st and 2nd month follow-up. Statistically significant differences were observed in LH levels before and after the PRP administration in the groups A (*p* < 0.05) and B (*p* < 0.001). (**C**) E2 of all groups prior to treatment, after the 1st and 2nd month follow-up. Statistically significant differences were observed in E2 levels before and after the PRP administration in all groups (*p* < 0.001). (**D**) AMH of all groups prior treatment, after the 1st and 2nd month follow-up. Statistically significant differences were observed in AMH levels before and after the PRP administration in groups A (*p* < 0.01) and B (*p* < 0.001). * *p* < 0.05, ** *p* < 0.01, *** *p* < 0.001.

For the last group which also included menopausal participants, a total of 19 women met the inclusion criteria and finally enrolled (Figure 7). In the same way, as the aforementioned groups, the levels of FSH and LH declined after the autologous PRP infusion, whereas the levels of E2 and AMH were elevated (Table 6 and Figure 8). Besides the initial high levels of FSH and LH, a remarkable reduction of more than 50% in both parameters were detected after the PRP infusion. In addition, in 2 out of 19 women the return of the menstrual cycle was reported. Ultrasound proofs regarding the beneficial effect of the autologous PRP administration are provided as Supplementary Materials.

### 3.3. Proposed Model of Ovarian Rejuvenation Using the Autologous PRP

**Figure 9:**
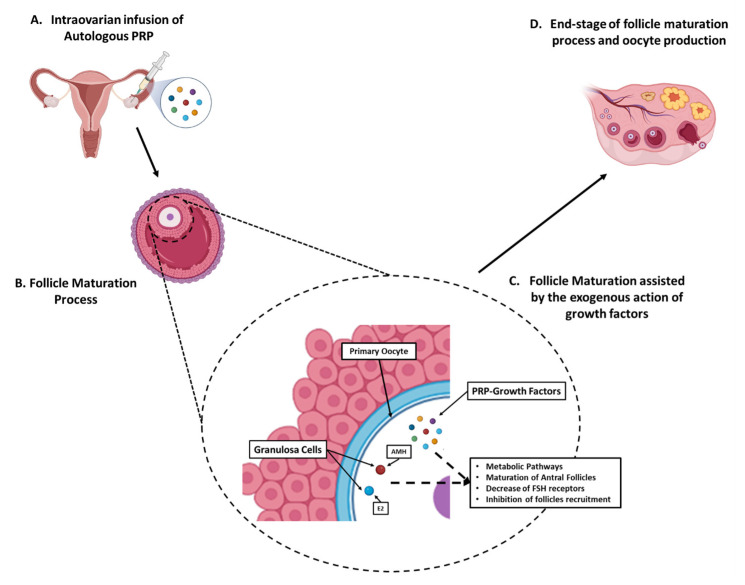
Proposed model of intraovarian rejuvenation utilizing the autologous PRP. (**A**) Initially, the ultrasound-assisted intraovarian administration of autologous PRP was performed to the patients of all groups. (**B**) The PRP-contained growth factors can positively influence the follicle maturation process. (**C**) During the preantral and antral stages, the PRP-growth factors e.g., PDGF, TGF-β, VEGF, HGF, IGF, and EGF can assist in hormone balance restoration, through implication to metabolic pathways, hence resulted to increase in AMH and E2 levels and in parallel the decrease in FSH and LH levels. Moreover, the increase in AMH results to further follicle maturation, decrease in FSH receptors and inhibition of follicle recruitment from the resting reservoir. (**D**) Finally, the antral follicles successfully maturated and ovulated, resulting to the end-stage follicle maturation process with the production of a functional oocyte.

Based on the results of our study, we could propose a model to better describe ovarian rejuvenation mediated by the exogenous PRP administration. As is already known, PRP is rich in growth factors, such as PDGF, TGF-β, VEGF, HGF, IGF, EGF and others, that can influence, in a positive way, follicle maturation, ovulation and oocyte production. The intraovarian infusion of PRP in all groups restored the hormone balance between FSH, LH and AMH, E2, which further resulted in successful conception and live births. Interestingly, in group E (women with menopause) after the PRP administration, the return of the menstrual cycle was also reported. Hence, the proposed therapeutic approach driven by the PRP intraovarian administration is in detail presented in Figure 9.

## 4. Discussion

The intraovarian injection of PRP can assist in the improvement of ovarian function in women with POR, POI or at perimenopause and menopause stages [32,33,34,35]. One of the main challenges of reproductive medicine is to rejuvenate ovarian function to increase the number of released follicles [36]. Considering this, PRP administration may assist in ovarian rejuvenation, exerted by the action of growth factors, and the current pilot study focused on the ovarian function assessment after the autologous PRP injection.

In the current study, a total of 582 participants aged 22–56 years were diagnosed with POR, POI or were at perimenopause and menopause stages. From those, a total of 253 women finally met the inclusion criteria and enrolled in the study. To date, this is the first study that includes a great number of participants, compared to previously conducted studies worldwide [32,33,34,35]. Based on the ovarian dysfunction type and in correlation with the average age, participants were divided into five groups. No side effects or severe pain were reported by any of the participants during the intraovarian PRP injection or within the two-month follow-up, suggesting that the proposed treatment is safe and well-tolerable.

Moreover, all participants experienced an improvement in ovarian function, which was further confirmed by changes in their hormonal profile, successful pregnancy and live births achievement or menstrual cycle return. Based on our results, the intraovarian PRP injection resulted in a progressive increase in E2 and AMH levels, while in parallel, the levels of FSH and LH were decreased. Specifically, considering group A, statistically significant differences were observed in all parameters (FSH, LH, E2 and AMH), followed by successful pregnancies and live births in 28% of the participants with POR or POI. In the same way, participants of group B presented a dramatic decrease in FSH and LH levels and an increase in E2 and AMH levels. In this cohort, 18% of the participants reported the return of their menstrual cycle and 13% successfully conceived. On the other hand, PRP administration in participants of the remained groups (C, D and E) presented differences in FSH and LH levels (decrease) and E2 and AMH levels (increase), although great discrepancies in the above levels within the groups were observed. The observed variability in the analyzed parameters can be explained partly by the fact that the age of the participants in these groups was significantly higher compared to groups A and B. However, the return of the menstrual cycle and nine pregnancies accompanied by live births were reported. Moreover, the results of the current study were further confirmed by those presented in the study of Pantos et al. [37]. Specifically, Pantos et al. had a total of 120 participants which they classified into POR, POI, perimenopause and menopause groups. In this study, the majority of the participants exhibited a significant reduction in FSH and LH levels and an increase in E2 and AMH levels, similar to our results. Moreover, our study included a higher number of participants compared to the study of Pantos et al., however, this can further confirm the beneficial effect of the intraovarian infusion of autologous PRP [37]. Currently, more than 14 clinical trials, deposited in the world database (www.clinicaltrials.gov, accessed on 25 October 2022) are performed, evaluating the safety, tolerability and beneficial outcome of the PRP obtained either from the autologous or allogeneic origin [38].

The results provided herein showed that the PRP-derived growth factors can implicate the restoration of the pathologic mechanism of ovarian dysfunction [33,34,35]. It is well known that PDGF, TGF-β, VEGF, HGF, IGF, EGF and others contained in PRP have a positive impact on tissue regeneration and wound healing [39]. Indeed, PDGF, besides the impact on cell proliferation and migration, alongside VEGF and TGF, plays a crucial role in angiogenesis and the neo-angiogenesis process [40]. It has been shown that in women suffering from POR or POI, the molecular network promoting angiogenesis has been significantly disrupted, and this phenomenon may further contribute to the impairment of ovarian functionality [41]. In this way, the administration of autologous PRP, which contains a high concentration of PDGF, VEGF and TGF-β may potentially activate the endothelial cells in order to activate the neo-angiogenesis procedure, thus positively affecting the ovarian function [40]. It is speculated that the neo-angiogenesis promoted by growth factors, such as PDGF, TGF-β and VEGF, may enhance the ovarian environment to support the small secondary pre-antral follicles growth, which will result in the production of large, ovulatory antral follicles [40,42,43].

Additionally, in our study after the intraovarian PRP administration and after a two-month follow-up, we observed restoration of hormone levels in most of the women of all groups. This may further suggest that the contained growth factors, besides the impact they may have stimulating specific intracellular signaling pathways (e.g., proliferation, migration, protein secretion), are suggested to positively affect the Hypothalamus Pituitary Adrenal (HPA) axis, mostly within the first month after the PRP administration [44]. This phenomenon is further confirmed by other researchers in the field, however, the exact mechanism by which PRP can affect the HPA axis sensitivity has not been well clarified yet [37]. More experimental procedures involving the use of animal models, in order to obtain representative tissue biopsies, are required to be performed, to acquire safe conclusions. Moreover, it has recently been shown that PRP, besides the growth factors, is rich in immunomodulatory molecules such as the Hepatocyte Growth Factor (HGF), indoleamine 2,3 dioxygenase, galectins and others [45]. These molecules can potentially tolerate acute immune responses by modifying overactivated immune cells such as M1 macrophages, Th1 cells, B lymphocytes and dendritic cells (DCs). Importantly, it has been shown that the immune response shifting is mediated through the paracrine effect of HGF, IDO and galectins, enhancing in this way the adaptation of anti-inflammatory properties (through the phenotype switch of immunity cells) and the apoptosis/necrosis of overactivated T and B cells [45]. For this purpose, currently, the PRP has been considered an important source for eye drops production, which can be used in patients suffering from dry eye as a result of Sjogren’s syndrome [45]. In this way, and considering that female infertility is closely related to an established acute inflammatory microenvironment, as a consequence of the presence of autoimmune disorders (e.g., Thyroid autoimmunity) or idiopathic reasons, the administration of PRP may have a beneficial effect in terms of regulating acute inflammatory responses. It has been published in the literature that 5% of all spontaneous POI cases are due to autoimmunity and acute inflammatory responses [46,47]. In this way, one of the reasons for ovarian rejuvenation in these patients may be the mediated anti-inflammatory responses of the immunomodulatory molecules contained in PRP.

Moreover, the beneficial outcome of PRP administration was also noted in participants of advanced ages (at pre-menopause or menopause stage). After the autologous PRP administration, the return of the menstrual cycle was reported in 15% of women aged above 48 years old, accompanied by two complication-free pregnancies. Our results further supported that the autologous PRP treatment increased the number of follicles, which further led to the restoration of the hormonal profile and return of the menstrual cycle. As has been previously mentioned, PRP is considered safe and free of adverse effects compared to the classical hormonal (HR) treatment [37,48,49]. Importantly, when there is a high risk of cardiovascular disease (CVD), the administration of HR is strongly considered a contradiction [37,48,49]. On the other hand, there is no currently known contradiction regarding PRP administration in patients suffering from CVD.

Considering the data presented in this study, the intraovarian administration of autologous PRP is considered an effective alternative treatment in the majority of participants. Women aged < 45 years are considered to be the best responders, while also the PRP administration seems to exert a beneficial effect in women with advanced age. Autologous PRP administration is a safe and tolerable alternative treatment. However, its production must be performed by a licensed laboratory with highly trained personnel, while its administration should be performed by a well-trained physician, in order to acquire the minimum risk. Moreover, overall, the PRP treatment should be performed in accredited clinical centers after acquiring the approval of the Bioethics Committee (at the local or international level). PRP production and administration fall into the terms of “minimally manipulation”, where the whole procedure involves only the centrifugation and PLTs isolation from the autologous peripheral blood, thus this therapy can be performed in private or public health care units [50]. Another parameter that should be considered during the PRP production process is the optimum number of isolated PLTs. In our study, the average number of isolated PLTs after the centrifugation steps was >1180 × 10^6^. However, currently, different PRP protocols and commercial kits exist, which can result in great variabilities in PLT isolation among the different research groups worldwide [51,52]. Additionally, it should be pointed out that besides the PLT number, the growth factor concentration should be determined, in order to correlate better the effect of PRP on ovarian function.

Besides the encouraging results which were presented here, this study is characterized by several limitations. Compared to other studies, here, no classifications between responders and no responders after the PRP administration were performed. Especially in groups D and E, higher standard deviations were presented compared to groups A to C. Additionally, no presence of control groups in each category nor the determination of the growth factor content of the produced PRP was performed. The relatively limited time of the follow-up can also be considered a further limitation. However, compared to the already published studies, herein a great number of participants (253) were enrolled, thus the extracted results are considered highly valuable for researchers in the field. Currently, a second study is being prepared, enrolling a greater number of participants, where the classification into responders and non-responders will also be taken into account. In parallel, another study is being designed using properly infertile animal models, in order to assess the safety, tolerability, and ovarian function improvement after autologous or allogeneic PRP infusion. Furthermore, proper biochemical examinations and tissue biopsies from animal models will be acquired, shedding further light on the effect of PRP on the HPA axis.

Notably, recent studies exhibited the beneficial effects of PRP also to endometrial reconstruction. The success of embryo implantation is closely related to endometrial thickness. A pilot study contracted by Zadehmodarres et al. [53] presented data reflecting the endometrium thickness increase after the autologous PRP administration. These results were further confirmed by others, suggesting that the combined application of intrauterine and intraovarian injection of autologous PRP, could result in a thickness increase (>9 mm), thus becoming more efficient for women to conceive [54,55,56,57].

Infertility (both male and female) has a great socioeconomic impact and psychological burden on candidate parents. In addition, compared to men, women exhibited more stressful experiences, especially when they had difficulties in conceiving after scheduled sexual intercourse [58,59]. Moreover, in the case of POI, it is estimated that >1% of women by the age of 40 years are affected by this condition, and for this reason, several therapeutic protocols are designed and evaluated [60]. However, these treatments are not always successful, and most times oocyte donation, surrogacy and adoption are the only available options in order for motherhood to be achieved. This fact is not always acceptable to a number of candidate parents who desire to have genetically related offspring. Intraovarian PRP administration appears to be considered as an alternative encouraging protocol for those women, however, a greater number of studies are needed to be performed before acquiring safe conclusions.

To date, concerns regarding the PRP intraovarian administration may logically arise. However, the proposed strategy targeting ovarian rejuvenation is based on the preparation and use of autologous PRP. Hence it may be considered that autologous PRP is safe, and mostly is devoid of transmittable diseases such as HIV, hepatitis, West Nile virus and Cruetzfeldt–Jacob disease. Notably, the latter transmittable agents are still considered a major issue when there is a need for blood or blood product transfusion. Furthermore, PRP shares common characteristics with blood such as pH, lactate, glucose, PCO_2_, PO_2_, PT and APTT, and therefore cannot induce any further biochemical imbalances at the time of administration. In addition, specific care must be applied during its preparation procedure to avoid bacterial contamination and growth, which may impair its application. Moreover, and considering the safety and tolerability of the intraovarian PRP infusion, during the two-month follow-up, no adverse reactions were reported by all participants. Currently, there is no evidence in the literature that autologous PRP injection, which is rich in key growth factors for ovarian rejuvenation, can further induce or enhance ovarian damage and cancer. Although there is no such association between the proposed therapeutical strategy and side effects occurring in the reproductive system, extensively evaluation for longer periods must be performed in order to assure the patients’ long-term safety. Based on the encouraging results of this pilot study, other studies are now being designed also considering the short and long-term safety of the patients.

In addition, PRP protocols can be further enhanced by the combined topical application of Mesenchymal Stromal Cells (MSCs) derived from the autologous lipoaspirates [61]. MSCs are considered key drivers of tissue regeneration and immune regulation, due to their differentiation abilities and paracrine effects and currently, these unique stem cells are applied in a great number of studies focused on the restoration of ovarian function [62,63,64].

## 5. Conclusions

In conclusion, the administration of autologous PRP can be considered a safe and tolerable alternative approach to the classical ones for ovarian rejuvenation. However, the production process, the administration and the follow-up should be strictly monitored and performed by accredited clinical centres ensuring the highest safety level for the participants. To provide further evidence for the ovarian function benefit outlined by the PRP administration, our research is currently well-designing a randomized clinical trial, including a greater number of participants in POI, POR, perimenopause and menopause stages, which will be further licensed by the Ethics Board of Centre of Human Reproduction and submitted to the clinical trials database (www.clinicaltrails.gov, accessed on 25 October 2022). The research for the proper management of ovarian insufficiency represents the “holy grail” in the field of reproductive medicine, and in this way the administration of autologous PRP appears to be an effective “off-the-shelf” therapeutic treatment for women with ovarian dysfunction. Performing more research studies focused on the evaluation of this alternative therapy, the acquired data will better reflect the beneficial use of PRP in gynecology.

## Figures and Tables

**Table 1:** Detailed description of participants’ characteristics, which were included in the randomized observational pilot study.

Groups	Eligibility Criteria	Standard Examination before the PRP Intraovarian Infusion	Follow-Up Monitoring	Assessment of the Outcome after the PRP Infusion
A (*n* = 100)	Age: 22–38 years POR or POI	Determination of FSH, LH, AMH and E_2_ levels	Performed at 1st and 2nd month	Determination of FSH, LH, AMH and E2 levels Successful Pregnancy: Y/N After Natural Conceive Or IVF Cycle Or ICSI-ET Cycle
B (*n* = 200)	Age: 39–44 years POI Perimenopause			
C (*n* = 160)	Age: 45–57 years Menopause			
D (*n* = 160)	Age: 48–50 Menopause			
E (*n* = 62)	Age: 51–56 Menopause			

POR: Poor Ovarian Response; POI: Premature Ovarian Insufficiency; FSH: Follicle Stimulating Hormone; LH: Luteinizing Hormone; AMH: Anti-Mullerian Hormone; E_2_: Estradiol; PRP: Platelet Rich Plasma; ICSI: Intracytoplasmic Sperm Injection; ET: Embryo Transfer.

**Table 2:** Evaluation of the Intraovarian PRP infusion in group A (POR or POI participants). Statistically significant differences regarding the levels of FSH (*p* = 0.001), LH (*p* = 0.046), E2 (*p* = 0.035) and AMH (*p* = 0.012), were observed after the PRP infusion.

Participant’s Characteristics		Biochemical Parameters	Prior Treatment	Month 1 Follow-Up	Month 2 Follow-Up	*p*-Value
*n*	25	FSH (IU/mL)	67.15 ± 8.72	22.74 ± 4.75	14.17 ± 2.93	0.001
Age	29.8 ± 3.9	LH (IU/mL)	27.51 ± 5.13	15.42 ± 3.42	12.25 ± 4.21	0.046
Number of Pregnancies	6	E_2_ (pg/mL)	72.78 ± 10.42	152.05 ± 8.73	160.02 ± 9.85	0.035
Number of Live Births	6	AMH (ng/mL)	0.23 ± 0.04	0.50 ± 0.03	0.75 ± 0.08	0.012

**Table 3:** Evaluation of the Intraovarian PRP infusion in group B (POI). Participants of group B presented a statistically significant decrease in FSH and LH (*p* < 0.001) and an increase in E2 and AMH (*p* < 0.001).

Participant’s Characteristics		Biochemical Parameters	Prior Treatment	Month 1 Follow-Up	Month 2 Follow-Up	*p*-Value
*n*	100	FSH (IU/mL)	35.52 ± 9.53	16.85 ± 4.86	10.58 ± 3.42	<0.001
Age	42.1 ± 2.1	LH (IU/mL)	18.64 ± 4.31	12.42 ± 8.87	7.38 ± 1.58	0.001
Number of Pregnancies	28	E_2_ (pg/mL)	50.45 ± 5.62	100.68 ± 12.85	114.04 ± 5.63	<0.001
Number of Live Births	28	AMH (ng/mL)	0.66 ± 0.11	0.87 ± 0.04	1.42 ± 0.07	<0.001

**Table 4:** Evaluation of the Intraovarian PRP infusion in group C (menopause). Statistically significant differences were observed regarding the levels of FSH and E2 (*p* < 0.001) in the participants of the current group.

Participant’s Characteristics		Biochemical Parameters	Prior Treatment	Month 1 Follow-Up	Month 2 Follow-Up	*p*-Value
*n*	55	FSH (IU/mL)	40.85 ± 7.24	29.91 ± 3.55	22.35 ± 2.81	<0.001
Age	45.8 ± 0.7	LH (IU/mL)	20.91 ± 2.25	18.02 ± 2.48	17.10 ± 2.36	<0.001
Number of Pregnancies	7	E_2_ (pg/mL)	70.46 ± 9.73	84.55 ± 4.56	143.26 ± 8.75	<0.001
Number of Live Births	7	AMH (ng/mL)	0.34 ± 0.05	0.46 ± 0.05	1.20 ± 0.04	0.656

**Table 5:** Evaluation of the Intraovarian PRP infusion in group D (menopause). A statistically significant difference was observed in the levels of E2 (*p* = 0.003) after the PRP infusion and 2 months follow-up.

Participant’s Characteristics		Biochemical Parameters	Prior Treatment	Month 1 Follow-Up	Month 2 Follow-Up	*p*-Value
*n*	54	FSH (IU/mL)	50.59 ± 5.64	47.81 ± 8.85	42.95 ± 9.84	0.514
Age	48.3 ± 1.6	LH (IU/mL)	23.09 ± 1.98	18.43 ± 7.26	18.03 ± 7.75	0.869
Number of Pregnancies	2	E_2_ (pg/mL)	77.71 ± 9.90	113.12 ± 10.84	116.95 ± 18.45	0.003
Number of Live Births	2	AMH (ng/mL)	0.36 ± 0.04	1.37 ± 0.07	1.54 ± 0.07	0.618

**Table 6:** Evaluation of the Intraovarian PRP infusion in group E (menopause). A statistically significant difference was observed regarding the levels of E2 (*p* = 0.002) after the PRP infusion and 2 months follow-up.

Participant’s Characteristics		Biochemical Parameters	Prior Treatment	Month 1 Follow-Up	Month 2 Follow-Up	*p*-Value
*n*	19	FSH (IU/mL)	75.91 ± 4.21	49.56 ± 9.68	41.07 ± 10.76	0.319
Age	51.61 ± 2.64	LH (IU/mL)	36.13 ± 4.04	32.47 ± 7.56	24.62 ± 8.29	0.773
Number of Pregnancies	-	E_2_ (pg/mL)	95.68 ± 5.32	126.11 ± 22.5	168.47. ± 25.09	0.002
Number of Live Births	-	AMH (ng/mL)	0.15± 0.08	0.25 ± 0.09	0.38 ± 0.15	0.860

## Data Availability

The data that support the findings of this study are available upon request from the corresponding author, Athanasios Garavelas. The data are not publicly available due to information that could compromise the privacy of patients.

## Supplementary Materials

The following supporting information can be downloaded at: https://www.mdpi.com/article/10.3390/medicines10030019/s1. Ultrasound proof for the beneficial effect of PRP.
