# Supplementary material for: Clinical Benefit of Autologous Platelet-Rich Plasma Infusion in Ovarian Function Rejuvenation: Evidence from a Before-After Prospective Pilot Study
Source: Medicines (Basel). 2023 Feb 27;10(3):19. doi: 10.3390/medicines10030019 (PMC10056078; doi:10.3390/medicines10030019)
Supplement: Supplementary file 1 [file medicines-10-00019-s001.zip › medicines-2043336-supplementary.pdf]

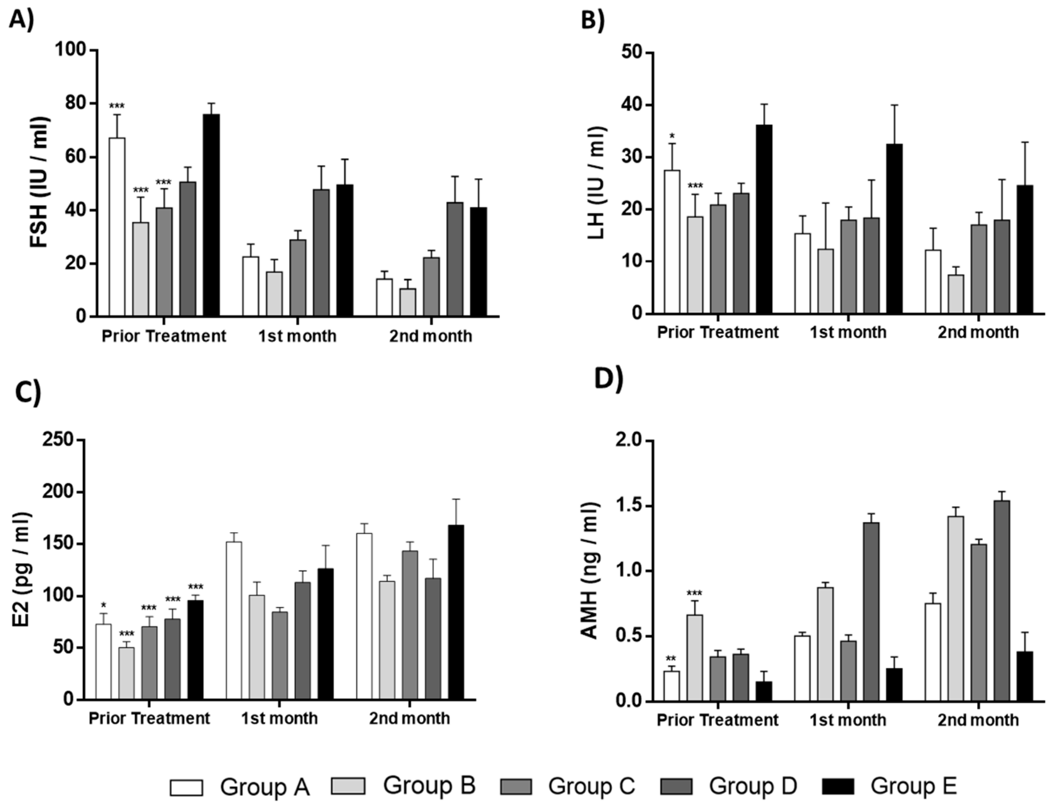

Figure S1. Representable diagrams regarding the levels of FSH, LH, E2 and AMH of the current pilot study. (**A**) FSH levels of all groups prior treatment, after the 1st and 2nd month follow-up. Statistically significant differences were observed in FSH levels before and after the PRP administration in the groups A ($p < 0.001$), B ($p < 0.001$) and C ($p < 0.001$). (**B**) LH levels of all groups prior treatment, after the 1st and 2nd month follow-up. Statistically significant differences were observed in LH levels before and after the PRP administration in the groups A ($p < 0.05$) and B ($p < 0.001$). (**C**) E2 of all groups prior treatment, after the 1st and 2nd month follow-up. Statistically significant differences were observed in E2 levels before and after the PRP administration in all groups ($p < 0.001$). (**D**) AMH of all groups prior treatment, after the 1st and 2nd month follow-up. Statistically significant differences were observed in AMH levels before and after the PRP administration in groups A ($p < 0.01$) and B ($p < 0.001$). * $p < 0.05$, ** $p < 0.01$, *** $p < 0.001$.
